# Effects of injured and dead cells of *Escherichia coli* on the colony‐forming rate of live cells

**DOI:** 10.1002/2211-5463.13051

**Published:** 2020-12-30

**Authors:** Mikako Saito, Norimasa Takatani, Tomonori Yoshida, Alvin Mariogani, Eol Cho, Hideaki Matsuoka

**Affiliations:** ^1^ Department of Biotechnology and Life Science Tokyo University of Agriculture and Technology Koganei Japan

**Keywords:** colony count method, dead cell, *Escherichia coli*, injured cell, osmotic stress, standard material

## Abstract

Osmotic stress‐induced injured cells of *Escherichia coli* were prepared by sorting live cells onto tryptic soy agar (TSA) containing 10–50% sucrose. The time course of colony‐forming rate (CFR%) was analyzed. A time delay in colony formation indicated a sublethal effect. The final CFR level at 24 h indicated the relative number of culturable cells irrespective of injury. A value of (100‐CFR)% at 24 h indicated a lethal effect. When cells were grown on TSA containing 10% sucrose, the time delay was 4 h and the lethal effect was 4%. However, dead cells inhibited the growth of live cells. Physical contact with insoluble matter derived from dead cells or dead cells themselves might have caused growth inhibition. These findings highlight a novel perspective on colony count methods in practical situations, such as when sampling foods containing a high concentration of sucrose.

## Graphical abstract

Single‐cell sorting of *Escherichia coli* was conducted to analyze the effect of osmotic stress–induced injury on the colony‐forming rate (CFR). Injury caused a time delay in the start of colony formation and a decrease in the final level of CFR. Surprisingly, dead cells also inhibited cell growth possibly due to the release of insoluble factors.

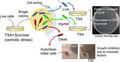

**Abbreviations**
CFDA: 6‐carboxyfluorescein diacetate
CFR: colony‐forming rate
FCS: forward scatter
SSC: side scatter
TSA: tryptic soy agar
TSA‐Suc: tryptic soy agar and sucrose
TSB: tryptic soy broth

Food and water products are sterilized to prevent microbial contamination, and its performance is checked by test methods that involve the measurement of the quantity of bacterial cells remaining in products. Test methods are based on a colony count using appropriate agar media. Globally validated reference methods are wholly based on the colony count method [1, 2]. The formation of a colony with a visible size is evidence that the original single bacterial cell was living. It is well understood that colony formation is influenced by various factors, such as medium composition, culture temperature, culture time, aerobic or anaerobic conditions, and sample‐derived coexisting substances. Occasions occur when that food sample‐derived substances might cause false‐positive or false‐negative results [3, 4]. These factors need to be carefully considered in practical tests.

A more difficult issue is the existence of injured bacteria [5, 6, 7, 8]. Freezing, drying, freeze‐drying, heating, γ‐radiation, and osmotic stress are treatments that might cause sublethal effects on bacterial cells. Even if the degree of injury is small, injured cells might not necessarily form colonies. Such cells might be regarded as nonculturable injured cells. When culture conditions are improved, however, such cells might recover from injury and grow to a colony of normal size with time. Such a case may be regarded as a false‐negative result. If the injury is serious, injured cells die. Such dead cells are believed to not affect colony count results. In this study, osmotic stress was selected as a cause of injury from among a variety of stresses.

Regarding the effects of dead cells, several factors released from bacterial cells might remain after their death to influence live bacterial cells. For example, autoinducers released by *Pseudomonas aeruginosa* were reported to be involved in quorum sensing [9, 10] and to suppress the growth of other bacterial cells. It was also shown that apoptosis‐inducing factors were generated by *Lactobacillus plantarum*, though their target was animal cells [11]. Lipopolysaccharides, important outer membrane components of Gram‐negative bacteria, induced a pro‐inflammatory stimulus in mammals [12]. Most bioactive compounds released from dead cells were active only on animal cells, except those relevant to quorum sensing. Moreover, in the case of proteins, these become inactivated during heat‐killing treatments. In fact, no report exists showing that dead bacterial cells directly influence the viability of live bacteria. Nevertheless, we suspect that dead cells or dead cell‐derived factors exert an effect on the colony‐forming rate (CFR) of live cells. In this study, autoclave‐killed cells of *Escherichia coli* were prepared to investigate the effect of dead bacterial cells on live bacterial cells.

## Materials and methods

### Microorganism

*Escherichia coli* ATCC8739, obtained from the American Type Culture Collection (Manassas, VA, USA), was precultured in tryptic soy broth (TSB; Becton Dickinson Co., Cockeysville, MD, USA); and its cell suspension was plated onto tryptic soy agar (TSA; Becton Dickinson Co., Cockeysville, MD, USA) and cultured at 37 °C.

### Preparation of injured *E. coli* cells by sorting cells directly onto TSA containing sucrose

Cell sorting was conducted according to a flow cytometric method as previously described [13, 14]. Briefly, live single‐cells of *E. coli* were live‐stained with 6‐carboxyfluorescein diacetate (CFDA) (Sigma‐Aldrich Japan, Tokyo, Japan). A plate, 86 mm in diameter, containing TSA and sucrose (TSA‐Suc plate) was set on an automatic stage installed in a cell sorter (FACSAria II; BD Co.). The automatic stage was used so that 100 cells could be sorted into a 10 × 10 grid pattern of spots at 1 cell/spot. The density of sucrose in TSA varied from 10% to 50%. During culture at 37 °C, the number of colonies was automatically recorded using a Biomatic™ S12 (MicroBio Corporation, Sendai, Japan). The threshold size of a colony was adjusted to 65 µm in diameter. The CFR was defined as 100 × *N*/*N*_0_ (%), where *N*_0_ was the number of sorted cells and *N* was the number of colonies. In this study, *N*_0_ was 100 and, therefore, CFR = *N* (%).

### Isolation of single injured *E. coli* cells

Every colony generated on a TSA‐Suc plate was thought to be a mixture of injured cells with different degrees of injury that were culturable and nonculturable injured cells. Therefore, it was necessary to isolate a cell fraction containing as high density of culturable injured cells as possible from the mixture. One colony was picked up from the TSA‐Suc plate, suspended in PBS (pH 7.0), stained with CFDA, washed with PBS, and then sorted on a TSA plate. The optimum sorting gate was determined based on a flow cytogram.

### Preparation of autoclave‐killed cells and live control *E. coli* cells

After culture of *E. coli* at 37 °C on TSA for 18 h, a colony was picked and resuspended in TSB for culture at 37 °C for 10 h with shaking at 170 r.p.m. The resulting culture was diluted with PBS, 0.1 m, pH 7.0, to adjust the cell density to 1.5 × 10^9^ cells per mL. Five milliliters of this cell suspension was transferred to a 50‐mL centrifugation tube. This tube was autoclaved at 121 °C for 15 min to prepare dead cells. The cell suspension just after autoclaving was defined as an original suspension of autoclave**‐**killed cells (Sample A1). This suspension was centrifuged at 15 000 ***g*** for 30 min. The resulting supernatant and precipitate were defined as a fraction of soluble factors derived from autoclave**‐**killed cells (Sample B) and a fraction of autoclave**‐**killed cell paste (Sample A2), respectively. *E. coli* cells were also cultured in TSB at 37 °C for 10 h with shaking at 170 r.p.m. and then diluted with PBS to adjust the cell density to about 10^4^ cells per mL. This cell suspension was defined as live control cells (Sample C).

### Co‐culture of live control cells and autoclave‐killed *E. coli* cells

Sample A1 was diluted with PBS to about 10^6^ cells per mL. Then dilution was carefully repeated to prepare a dilution sequence: 1/10, 1/10^2^, 1/(2 × 10^2^), 1/10^3^, 1/(2 × 10^3^), and 1/10^4^. The positive control was a sample containing only Sample C. The negative control was a sample containing only Sample A1. When the dilution rate of Sample A1 was 1, 1/10, 1/10^2^, or 1/10^3^, for instance, the ratio (the number of dead cells/ the number of live cells) was ~ 1000, 100, 10, or 1, respectively.

Sample A2 was suspended in PBS and the cell density was adjusted to about 10^6^ cells per mL. Then, its dilution sequence was prepared in the same way as Sample A1. Sample B was diluted with PBS to prepare the same series of dilutions as Sample A2. A 100 µL aliquot of Sample C was mixed with a 900 µL aliquot of Sample A1, A2, or B at respective dilution rates. A 100 µL aliquot of the mixture was plated onto TSA and incubated at 37 °C. The number of colonies was counted versus the dilution rates of Samples A1, A2, and B, respectively.

### Physical contact of live‐cell colonies with Sample A2 paste

*Escherichia coli* cells were cultured in TSB at 37 °C for 10 h and then diluted with PBS to adjust the cell density to 2.0 × 10^2^ cells per mL. Then, a 100 µL aliquot of the cell suspension was plated on TSA for culturing at 37 °C for 18 h. Four clumps of Sample A2 paste with a diameter of 0.5–1 mm were placed around a live‐cell colony with a diameter of 2–3 mm. Culturing was continued and colony growth observed.

### Measurement of pH of Samples A2 and B

The pH of Sample A2 was measured by placing a pH indicator paper onto the clump of cells. The pH of Sample B was measured by immersing a pH paper into the sample.

### Statistics

Specific details regarding statistical analyses are presented in the figure legends. In most cases, three test samples were analyzed per analytical item. Results are presented as mean ± SD. The statistical significance between two specific data groups was analyzed by paired two‐tailed Student's *t*‐test. The statistical significance of results is denoted by a *P*‐value or by marking with asterisk(s): ***P < 0.001, **P < 0.01, and *P < 0.05.

## Results

### Effects of sucrose density on properties of injured cells

**Fig. 1:**
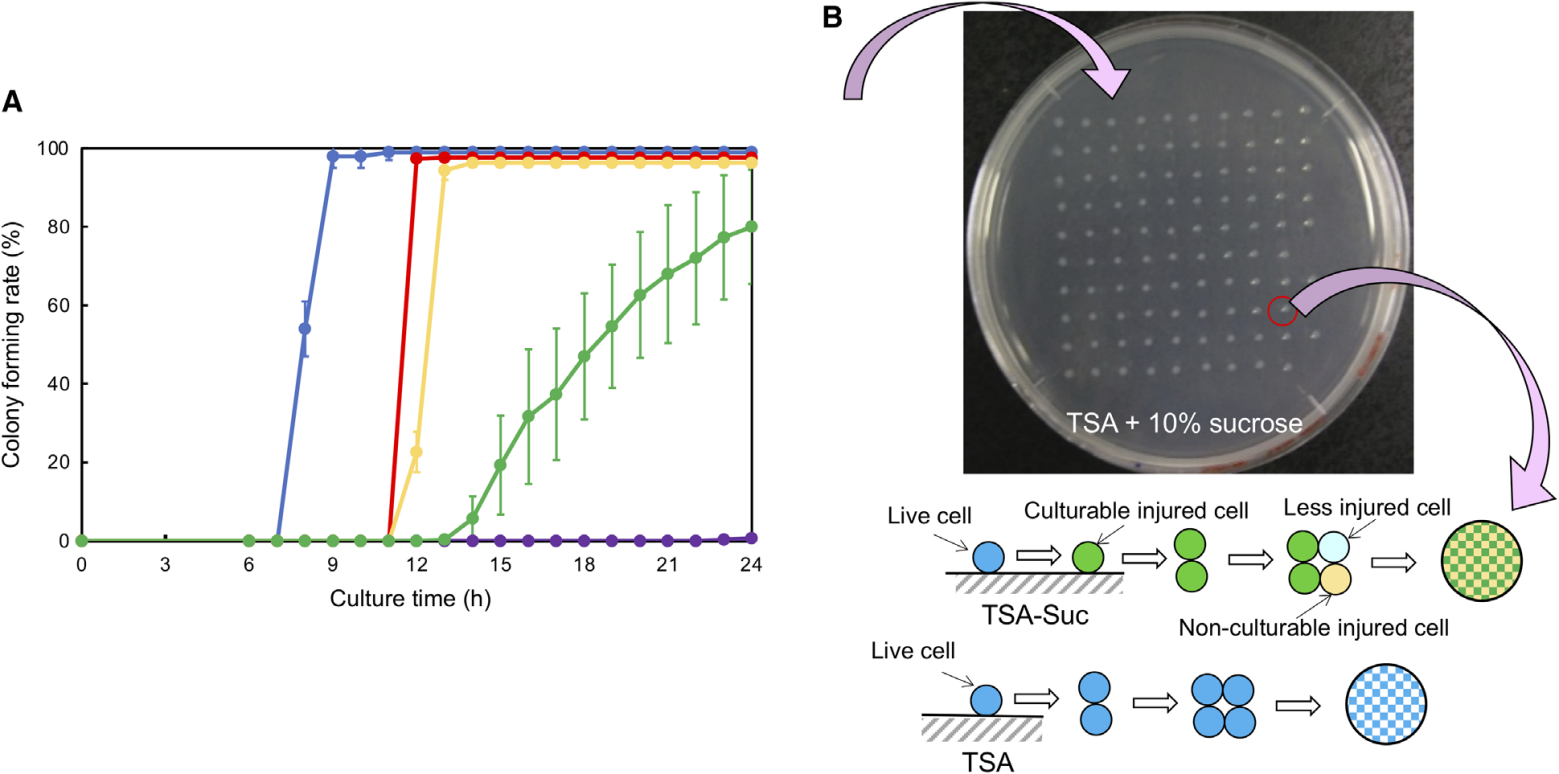
Effects of sucrose density on *E. coli* colony formation. (A) Time course of the number of *E. coli* colonies precultured in TSB and then grown on TSA with various percentages of sucrose: 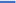 TSA, 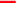 TSA + 10% sucrose, 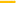 TSA + 20% sucrose, 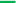 TSA + 30% sucrose, 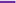 TSA + 50% sucrose. mean ± SD for *n* = 3. (B) A 10 × 10 pattern of colonies prepared by the sorting of live single‐cells. Live cell colonies: sorted onto TSA, Injured cell colonies: sorted onto TSA‐sucrose (Suc). Each colony of injured cells was composed of culturable and nonculturable injured cells.

When a colony of live cells was grown on TSA, these showed a sharp increase in CFR between 7 and 9 h to reach a steady level of colony formation (99%; blue line; Fig. 1A). When TSA contained 10% sucrose, the CFR increase for a colony was delayed by about 4 h, reaching the highest level (96%) within 1 h (red line). When the sucrose density was 30%, the increase in CFR for a colony was delayed markedly (green line). In the presence of 50% sucrose, no CFR increase was observed (purple line).

Based on the time courses for CFR, we defined the delay in the time of a CFR increase to be an indicator of the degree of a sublethal effect, and the final level of CFR at 24 h to be an indicator of the degree of a lethal effect.

Each live single‐cell sorted onto TSA‐Suc was thought to become an injured cell (Fig. 1B). The degree of injury is postulated to depend upon the sucrose density. At 30% or lower density of sucrose, cells were maintained in a culturable state but cell division was retarded. During colony growth, culturable cells might have become nonculturable, or, conversely, developed into fewer injured cells due to their adaptation to osmotic stress. Therefore, each colony generated on TSA‐Suc was thought to be a mixture of such cells.

### Flow cytograms of live and injured cells

**Fig. 2:**
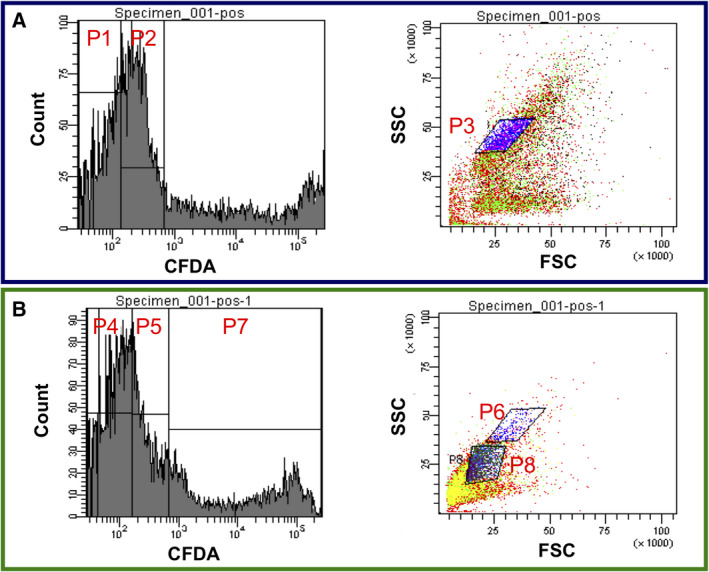
Comparison of flow cytograms of healthy and injured *E. coli* cells. (A) A healthy cell colony generated on TSA was suspended in PBS, stained with CFDA, and then sorted. (B) An injured but culturable cell colony generated on TSA‐Suc was suspended in PBS, stained with CFDA, and then sorted. Left panels: Fluorescence intensity of CF derived from CFDA indicates the viability of the cell based on intracellular esterase activity. Higher esterase activity leads to high CF fluorescence intensity and consequently higher cell viability. Right panels: FSC. A higher intensity indicates a larger cell size. SSC. A higher intensity suggests a higher complexity of intracellular structure and a more viable cell. A gating area for healthy cells was determined by the combination of (P1 or P2) and P3. A gating area for injured cells was determined by the combination of (P4, P5, or P7) and (P6 or P8).

Flow cytograms of live cells generated on TSA and injured cells generated on TSA‐Suc were compared (Fig. 2). Both forward scattering (FSC) and side scattering (SSC) of injured cells were lower than those of live cells. A low FSC indicated a small cell size and a low SSC suggested a low complexity of intracellular structure and a more viable cell. Therefore, osmotic stress‐injured cells were smaller and less viable than live cells.

### Properties of injured cell fractions containing different densities of culturable cells based on flow cytograms

**Fig. 3:**
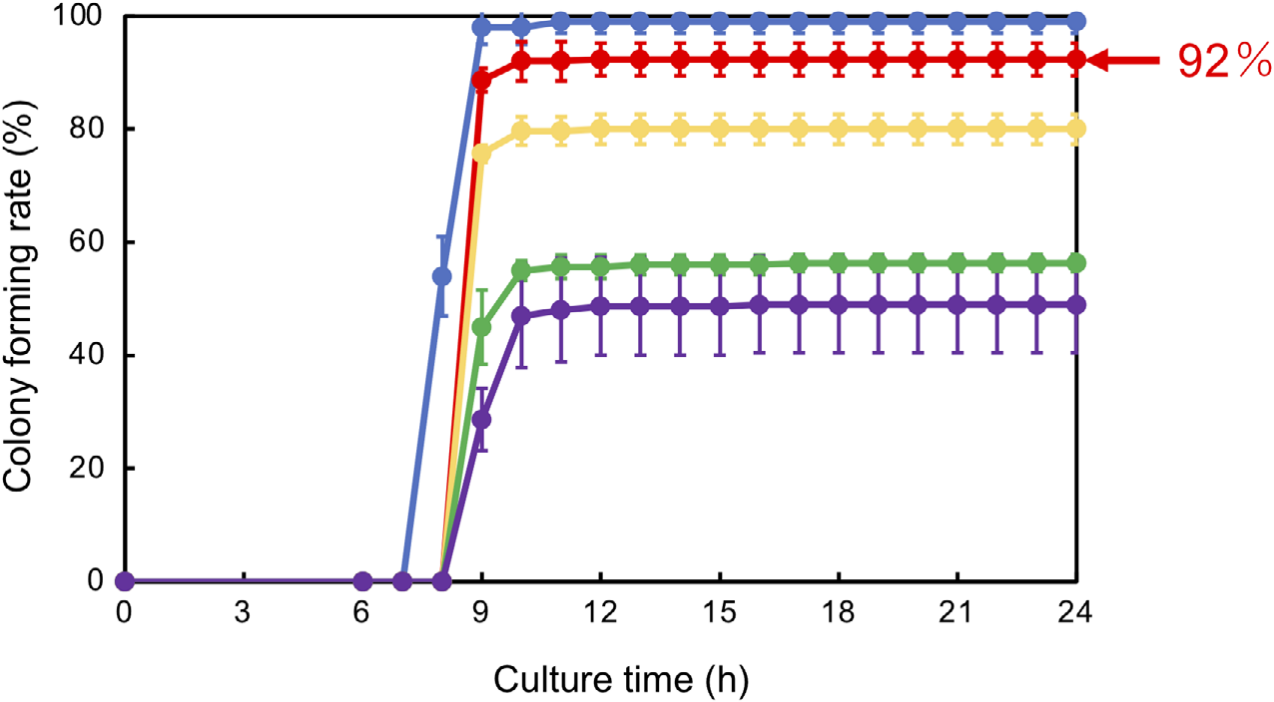
Colony‐forming processes for injured *E. coli* cells sorted from different gating areas. P2&P3 was selected as a gating area for healthy cells. Four gating areas P5&P6, P5&P8, P7&P6, and P7&P8 were selected as gating areas for injured cells. One hundred single cells were sorted from each gating area onto TSA. CFR% and (100‐CFR)% at 24 h were determined. 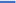 P2&P3 (control), 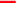 P5&P6, 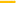 P5&P8, 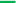 P7&P6, 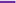 P7&P8. Mean ± SD for *n* = 3.

The gating area for live control cells was determined as P1&P2 according to a former study [14]. In contrast, for injured cells, four gating areas were selected based on flow cytograms. Each gating area was thought to contain culturable and nonculturable injured cells. One hundred single‐cells selected from P5&P6, P5&P8, P7&P6, and P7&P8 gating areas, respectively, were sorted onto TSA plates. In comparison with live control cells, the delay in time for the CFR increase was 1 h for every gating area (Fig. 3). In comparison with TSA‐Suc results (Fig. 1A), this delay in time was shortened from 4 to 1 h. Therefore, such injured cells might have partially recovered from osmotic stress‐induced injury during culture on TSA.

In comparison, the CFR final levels at 24 h differed between respective gating areas, indicating a difference in densities in nonculturable injured cells between gating areas. The cell fraction isolated from P5&P6 area showed a CFR of 92% at 24 h, the highest level. In this case, the density of nonculturable injured cells or dead cells was determined to be 8% [(100‐CFR)% at 24 h].

### No live cells in autoclave‐killed cell samples

A 100 µL aliquot of Sample A1 was plated on TSA and incubated at 37 °C for 55 h. No colony was generated (results not shown). Therefore, we conclude that no live cells remained in Sample A1.

### Effects of autoclave‐killed cells on the colony formation by live cells

**Fig. 4:**
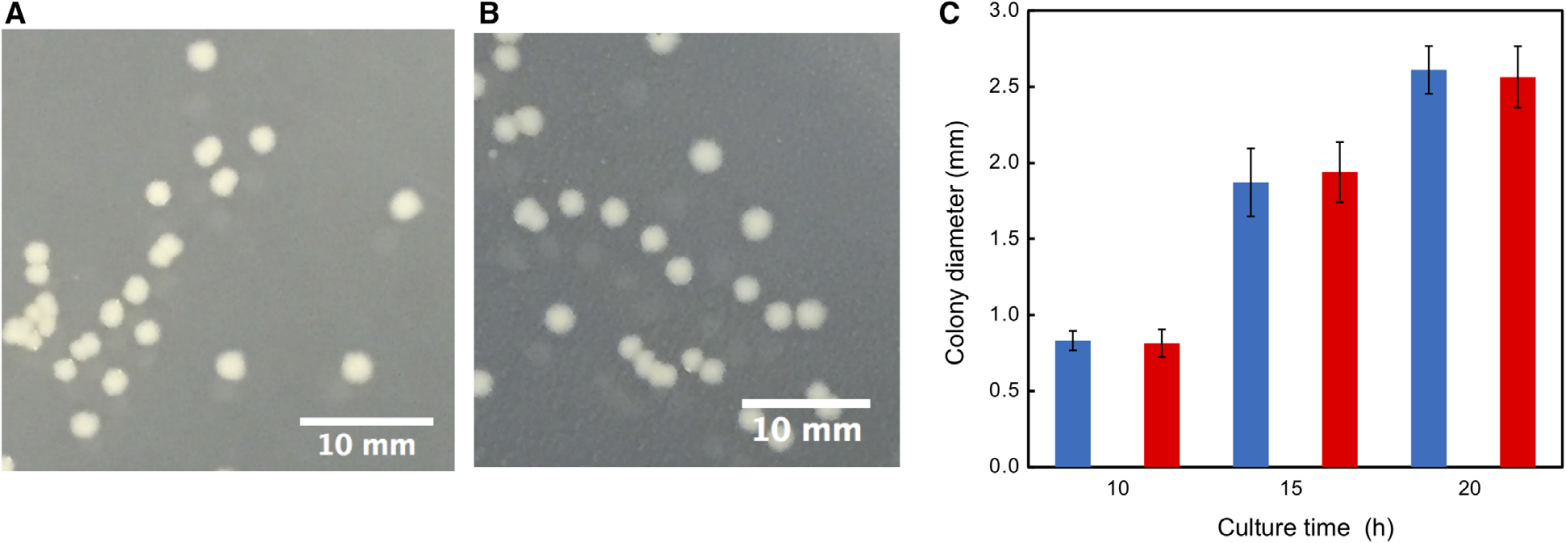
Size and shape of *E. coli* colonies formed during culture on TSA for 15 h in the absence and presence of autoclave‐killed cells. (A) Without dead cell. Bar scale = 10 mm. (B) With 1000 times more dead than live cells. Bar scale = 10 mm. (C) Colony size up to 20 h. 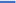: live cells without dead cells, 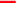: live cells with 1000 times more dead than live cells. Mean ± SD for *n* = 14.

**Fig. 5:**
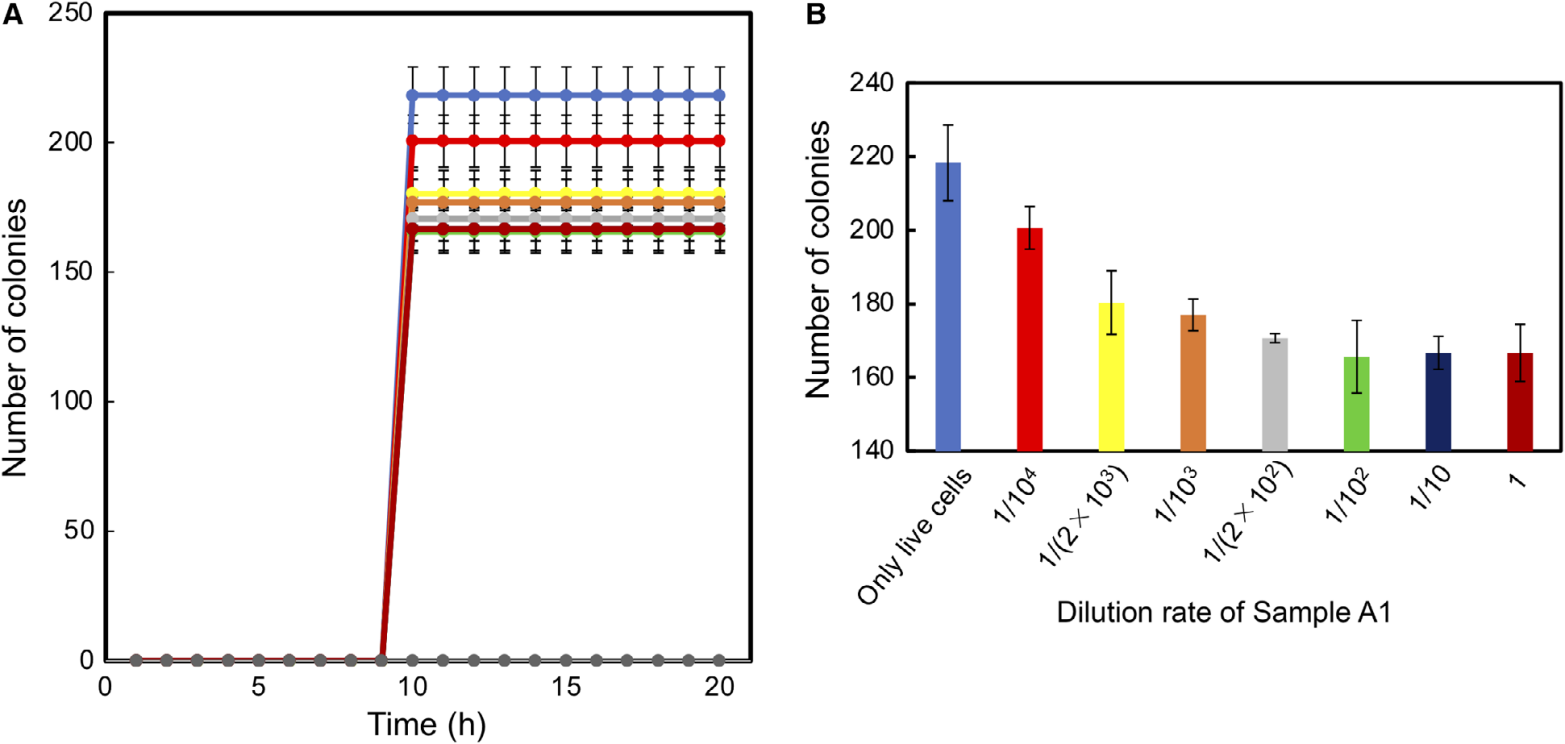
Effects of Sample A1 dilution rate on the number of *E. coli* colonies formed during culture on TSA for 20 h. (A) Time courses. (B) Colony count at 20 h. mean ± SD for *n* = 3. Dilution rates of Sample A1: 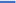 Only live cells, 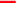 1/10^4^, 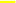 1/(2 × 10^3^), 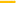 1/10^3^, 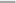 1/(2 × 10^2^), 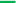 1/10^2^, 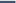 1/10, 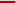 1, 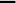 Only dead cells.

A mixture of Sample A1 and Sample C was plated onto TSA and cultured at 37 °C for 20 h. Colony size was observed and the number of colonies counted. We found that colony size was not influenced by the presence of autoclave‐killed cells (Fig. 4). The number of colonies, however, decreased as the density of Sample A1 increased (Fig. 5).

During autoclave treatment, *E. coli* cells are killed and intracellular components released. The soluble components might include active oxygen species and endotoxins while denatured cellular architecture might become insoluble clumps that adhere to live cells. We subsequently investigated whether colony formation was suppressed by such soluble and/or insoluble factors.

### Comparison of the effects of soluble and insoluble factors

**Fig. 6:**
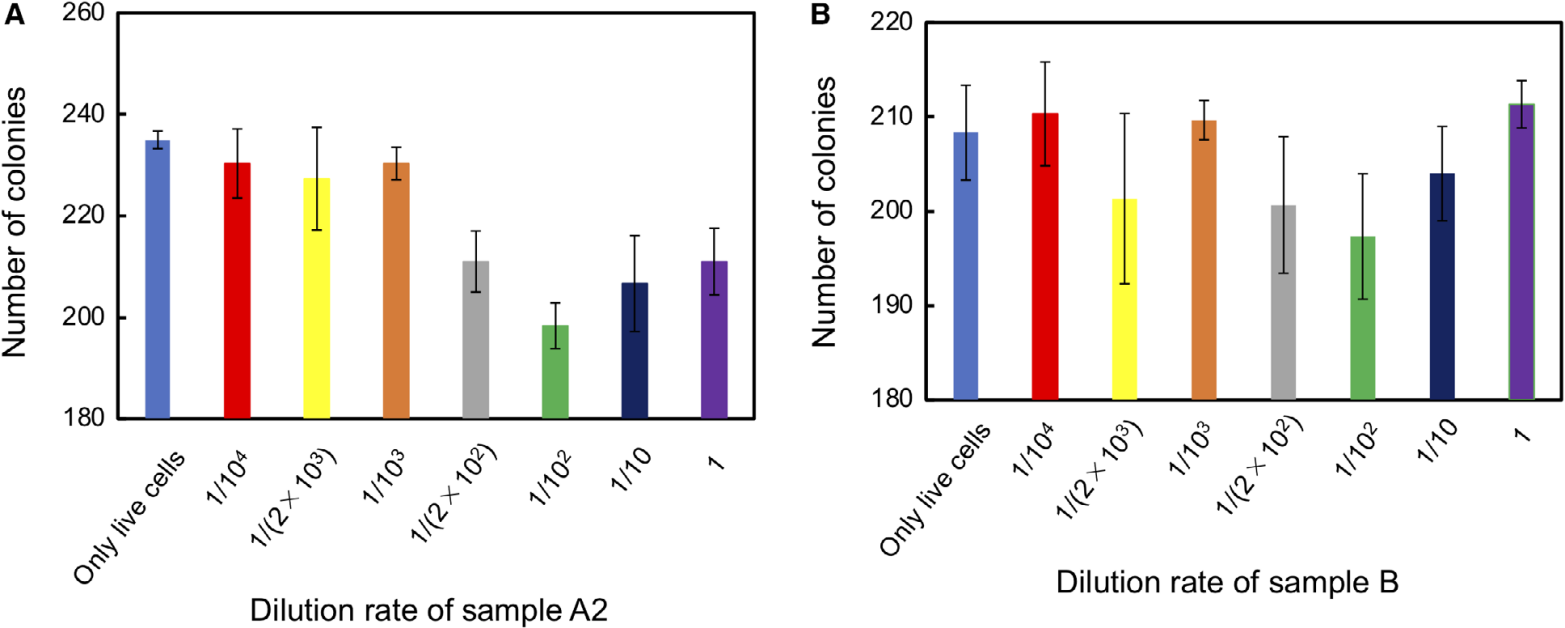
Effects of the dilution rates of Sample A2 and Sample B on the number of *E. coli* colonies formed during culture on TSA for 20 h. (A) Sample A2. Dilution rates of Sample A2: 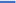 Only live cells, 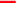 1/10^4^, 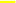 1/(2 × 10^3^), 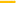 1/10^3^, 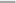 1/(2 × 10^2^), 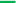 1/10^2^, 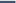 1/10, 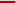 1, mean ± SD for *n* = 3. (B) Sample B. Dilution rates of Sample B: 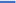 Only live cells, 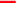 1/10^4^, 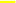 1/(2 × 10^3^), 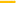 1/10^3^, 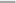 1/(2 × 10^2^), 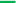 1/10^2^,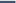 1/10, 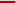 1, mean ± SD for *n* = 3.

A mixture of Sample A2 and Sample C was plated onto TSA and cultured at 37 °C for 20 h. The number of colonies decreased as the dilution rate of Sample A2 became 1/(2 × 10^2^) or lower (Fig. 6A). In contrast, Sample B had no effect on the number of colonies generated by Sample C (Fig. 6B).

This suggested that the suppression of colony formation was caused by insoluble factors.

### Colony growth behavior in the presence of autoclave‐killed cell clumps

**Fig. 7:**
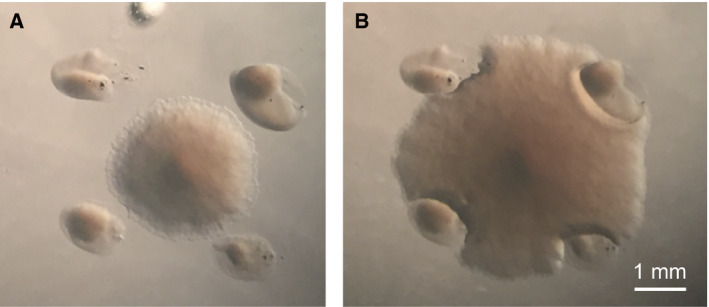
Colony growth in the presence of autoclave‐killed cell clumps. (A) Before contact of colony edges with autoclave‐killed cell clumps. (B) Cells cultured for 15 h after (A). Bar scale = 1 mm

After the placement of autoclave‐killed cell clumps around a colony of live cells, culturing was continued at 37 °C. The colony extended its edges in every direction while growing until it reached the clumps. Colony edges subsequently seemed to avoid the obstacles formed by these cell clumps (Fig. 7).

One possible reason was suspected to be obstacles created by the clumps so that the growing live‐cell colony was hindered from taking nutrients from the TSA. At the same time, however, the growing edge of the live‐cell colony seemed to be repelling those clumps after physical contact. The pH of the autoclave‐killed cell clumps was 6–7, and therefore, suppression of colony growth was not due to the surface pH of the clumps.

## Discussion

Injured cells are generated by a variety of stresses. The degree of injury depends upon the type and intensity of the stress. In real‐world test samples, however, it is difficult to determine the extent of injury of every contaminating bacterial cell. Therefore, for an accurate validation of test methods, it is necessary to evaluate beforehand effects on the colony count of every bacterial strain and sample matrix used (foods, feeds, or environmental samples). This study highlights an evaluation protocol for the case of *E. coli* and the use of a matrix of high osmotic stress.

The first step was an analysis of colony formation on TSA that contained sucrose. The sucrose density was varied for the quantitative analysis. Comparative results of CFR time courses (Fig. 1A) revealed that the effect of stress could be evaluated with respect to two points: a delay in a sharp increase in time of the CFR and finally reaching the CFR level. The delay in time indicated the degree of a sublethal effect and the final level of CFR at 24 h indicated the degree of a lethal effect. Conditions of 20% or less sucrose caused an ~ 4‐h delay as a sublethal effect but only showed a slight lethal stress (< 4% decrease in CFR). In contrast, 30% sucrose caused a 6‐h delay as a sublethal effect and a 20% decrease in CFR as a lethal effect. When the sucrose density was 50%, no colonies were generated indicating a 100% lethal effect.

The second step was the analysis of colony formation by isolated injured cells on TSA. Figure 3 revealed that injured cells generated a colony more rapidly on TSA than on TSA‐Suc, indicating a partial recovery from the initial injury level during culture. Such a result may simulate the practical case of a false‐negative result: a negative result is obtained during a regular test but colonies are generated during storage under less stressful conditions. The gating area of P5&P6 was found to be the optimum area where culturable injured cells were most densely present.

**Fig. 8:**
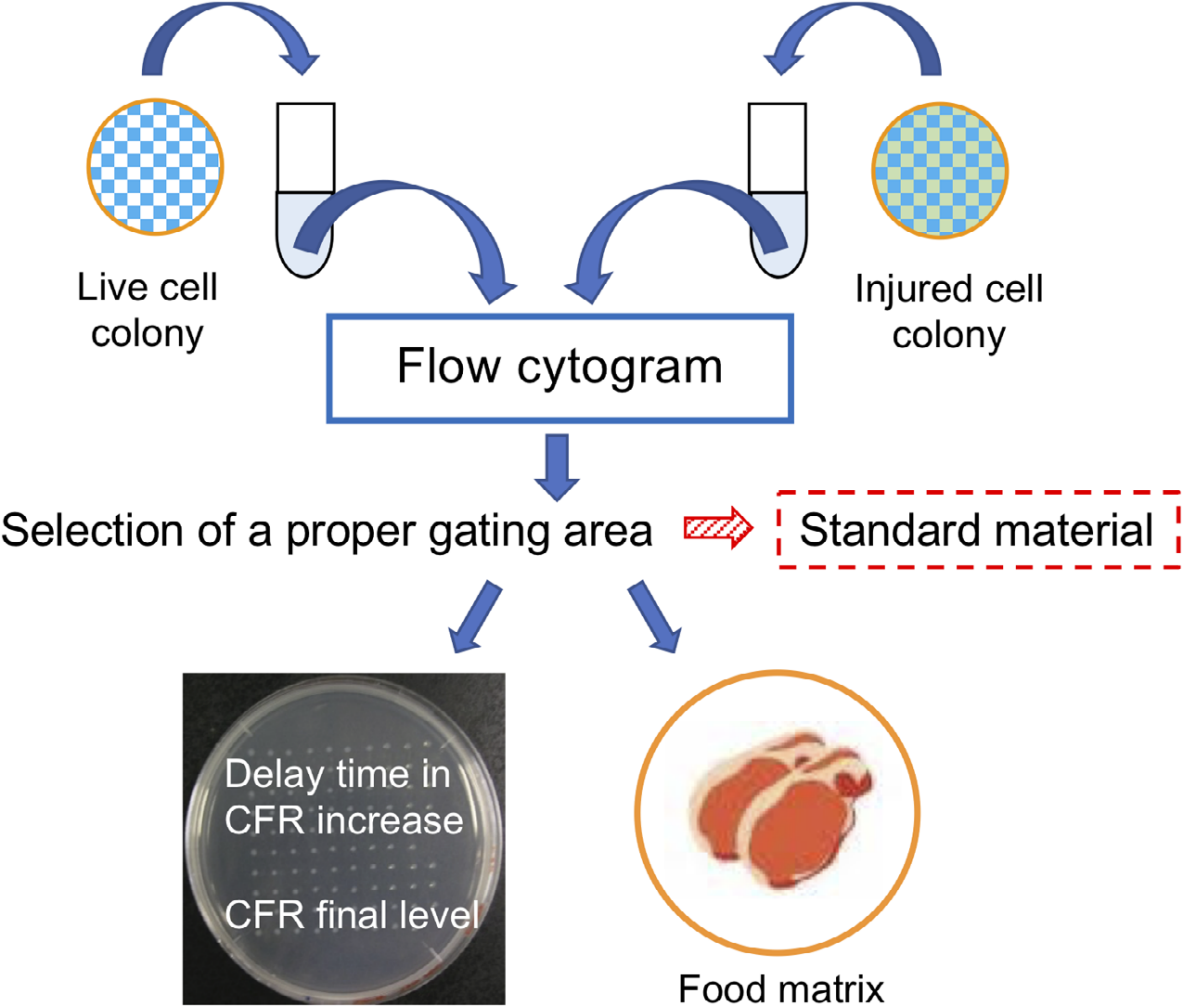
Flow cytometric preparation of a standard injured cells.

This two‐step analysis can provide a means for the preparation of standard material of injured cells with a specific degree of injury (Fig. 8). Once the optimum gating area, such as P5&P6, can be determined, single‐cells sorted from this area can be regarded as standard material. The injured single‐cells can be sorted onto TSA and practical samples in parallel. The properties of injured cells sorted onto practical samples can be verified later from the CFR properties observed on TSA. Therefore, making a database of optimum gating areas for various combinations of bacterial strains and practical samples would be of practical significance.

However, the effect of autoclave‐killed *E. coli* cells on the growth of live cells was surprising. Based on Figs 5 and 6, we suspect that physical contact with, or adsorption to, dead cells might have caused the growth suppression of live cells. The effect of soluble factors, if any, might have been small because of their low solubility or mucous property. A large gap was observed at the boundary of a live‐cell colony and an autoclave‐killed cell paste, suggesting retardation of the growth of the live‐cell colony. Such a retardation did not necessarily induce a lethal effect since the colony seemed to recover its original growth rate with time.

Assuming a physical‐contact hypothesis, it might be unsurprising that the decrease in number of colonies did not necessarily correlate with the dilution rate of dead cells (Fig. 5). Within a high concentration of dead cells [dilution rate < 1/10^2^, ratio of (the number of dead cells/the number of live control cells)> 10], these likely formed clumps might not have influenced live‐cell growth.

In conclusion, osmotic stress‐induced injury of *E. coli* cells caused a time delay in the initiation of colony formation and a decrease in the final level of CFR. Surprisingly, dead cells also inhibited cell growth possibly due to the presence of insoluble factors. These findings highlight a novel perspective on colony count methods in practical situations, such as foods containing a high concentration of sucrose.

Presently, however, it is unknown how to separate dead cells from test samples or to accurately evaluate their effects on colony counts would be the subject of a future study.

## Conflict of interest

The authors declare no conflict of interest.

## Author contributions

MS designed the study plan and wrote the manuscript. NT and TY performed the research and analyzed the data. AM and EC performed the research; and HM partially wrote the manuscript.

## Data Availability

The data are available from the corresponding author upon reasonable request.
